# The co-construction and emotion management of hope within psychosis services

**DOI:** 10.3389/fsoc.2023.1270539

**Published:** 2024-01-08

**Authors:** Patrick Brown, Amanda Scrivener, Michael Calnan

**Affiliations:** ^1^Department of Sociology, University of Amsterdam, Amsterdam, Netherlands; ^2^Retired, Maidstone, Kent, United Kingdom; ^3^School of Sociology, Social Policy and Social Research, University of Kent, Canterbury, United Kingdom

**Keywords:** co-construction, emotion management, hope, mental health services, phenomenology, psychosis, qualitative methods

## Abstract

**Introduction:**

There is a growing acknowledgement of the salience of hope for mental health service-users, in influencing care outcomes and recovery. Understandings of the processes through which hopes are co-constructed, alongside specific conceptualisations of experiences of hoping, remain limited however.

**Methods:**

This qualitative study explored how a range of stakeholders experienced and dealt with uncertainty within three purposively selected psychosis services in southern England. In this article we focus particularly on the co-construction of hope within participants' narratives and how this emotion work shaped experiences of hoping. In-depth interviews (*n* = 23) with service-users, professionals, managers and other stakeholders were analysed following a phenomenological approach.

**Findings:**

Hope was spontaneously identified by participants as a fundamental mechanism through which service-users and professionals managed uncertainty when vulnerable. Professionals were influential in shaping users' hopes, both intentionally and unwittingly, while some professionals also referred to managing their own hopes and those of colleagues. Such management of expectations and emotions enabled motivation and coping amidst uncertainty, for users and professionals, but also entailed difficulties where hope was undermined, exaggerated, or involved tensions between desires and expectations.

**Discussion:**

Whereas, hope is usually reflected in the caring studies literature as distinctly positive, our findings point to a more ambivalent understanding of hope, as reflected in the accounts of both service-users and professionals where elevated hopes were described as unrealistic and harmful, to the well-being of professionals as well as of service-users. It is concluded that a greater awareness within care contexts of how hopes are co-constructed by professionals and service-users, explicitly and implicitly, can assist in improving health care and healthcare outcomes.

## Introduction

This article contributes to a special issue focusing on the role of emotions in the context of healthcare. Hope, as a future-oriented emotion (Simpson, 2004), has increasingly been considered to be relevant to mental healthcare settings. Indeed a growing literature exists around the concept and role of hope, within health and social care contexts more generally and in contexts of supporting those with mental health problems more specifically, as acknowledged within earlier and more recent reviews (Cutcliffe and Koehn, 2007; Schrank et al., 2008; Wiles et al., 2008; Heller, 2014; Lohne, 2022). As a positive “emotional attitude” through which the hoper's subjective considerations and desires are oriented towards certain possible future outcomes (Simpson, 2004; Wiles et al., 2008), the significance of hope is apparent as follows: a means of coping with vulnerability and uncertainty in the present (Zinn, 2008; Brown and de Graaf, 2013); a tool for managing and alleviating anxiety in contexts where treatment proves ineffective (van Dantzig and de Swaan, 1978); motivating action and pursuit of future goals (Simpson, 2004; Lohne, 2022); and as a source of solidarity and mediation between those who may share common hopes (Rorty, 2002; Heller, 2014).

Growing interest in measuring hope and correlating factors within mental healthcare contexts (see Schrank et al., 2008) is often attributed to a recent focus on *recovery* (Bertolote and McGorry, 2005; Van Gestel-Timmermans et al., 2010), yet review articles denote a continuing paucity of adequate empirical research, ambiguous definitions (Cutcliffe and Koehn, 2007), and related problems of construct validity. A handful of studies acknowledge the influence of both professionals and service users in cultivating users' hopes in contexts of severe mental illness, including psychosis (Kirkpatrick et al., 1995; Darlington and Bland, 1999; McCann, 2002). There remains, however, a lack of in-depth case studies exploring how hope is created and managed amidst user-professional interactions (Cutcliffe and Koehn, 2007; Schrank et al., 2008; Wiles et al., 2008).

Conceptual studies denote an important tension between “hope-as-desire” for the less-than-likely and “hope-as-expectation” for the more probable (Simpson, 2004; Wiles et al., 2008; Brown and de Graaf, 2013). A dark side to hope correspondingly exists where “a longing to cope and to leave a position of vulnerability and/or despair may lead to mere possibilities being focused upon in a highly blinkered fashion” (Brown et al., 2014, p. 315). This has potential implications for the success of service users' recovery, for example, leading to avoidant coping strategies such as “sealing over” (Tait et al., 2004). Such potentially negative aspects of hope are largely missing within empirical research within mental health contexts (Schrank et al., 2008) and in wider research on hope, where hope is predominantly researched in terms of its benefits (Lohne, 2022) – being contrasted with hopelessness (Seligman, 1975) and related problems of suicide risk (Davidson et al., 2010; Heller, 2014) or “engulfment” within, and internalising of, labels such as schizophrenia (Cutcliffe and Koehn, 2007).

The positive emphasis given to hope within the psychiatric, mental health and related social sciences literature would seem to relate to the relative ubiquity of the recovery model as an approach to living with chronic psychosis-related conditions and, in turn, the prominent role of hope within this. Alongside its centrality, Ramon et al. (2007, p. 111) denote that this hope is not so much in living symptom-free but in a socially supported, agentic form of everyday coping and flourishing amidst interdependent support and the relative absence of disabling barriers such as stigma and exclusion. These same authors (Ramon et al., 2007, p. 110) locate these specific meanings around a hope-imbued recovery within a specific point in time, especially in the 1990s and early 2000s following the coinciding of the following: de-institutionalisation and community living; greater recognition of the experiential expertise of those living with psychotic symptoms; more emphasis placed on strengths of this group, rather than reducing people to their vulnerabilities; and growing acceptance of the social disability model of mental health problems.

Before the late twenteeth century, it was generally accepted that the “rule of thirds” (Harding et al., 1987) applied to the course of schizophrenia, whereby a third of those diagnosed could expect chronic illness, a third intermittent illness, and a third recovery (in a narrower, symptom-free sense). Hope in this context was that one would belong to the recovering cohort. The new framing of recovery broadened and changed the orientation of hope (from cured to coping), while qualitative studies of the personal experience of recovery (in a broader sense) identified “hope” as a key factor but the concept remained largely undefined (Saelor et al., 2014).

In the study described below, we drew on phenomenological traditions (Schutz, 1967; Smith and Osborn, 2003) to explore how participants understand experiences of, and their ways of coping amid, vulnerability and uncertainty, such as through relations characterised by (dis)trust and/or risk; though we aimed to remain open to a wide spectrum of different coping processes (Zinn, 2008). Hope emerged spontaneously within the accounts of service users, professionals, and other stakeholders. In contrast to much previous research, the analysis below explicitly considers the relevance of hope for both professionals *and* service users (McCann, 2002), to analyse how hope was, often implicitly (Schutz, 1967), co-constructed and managed within professional-user relationships and broader service contexts. This management of hope was an important and unforeseen finding in our analysis of the data in our study coded as pertaining to hope. In seeking to conceptualise considerations of hope as an emotion (Simpson, 2004) pointed us towards the relevance of Hochschild's (1979) work on emotion management, to which we turn in the next section. The central research questions in our analysis are as follows: through what processes was hope co-constructed by service users and professionals? And what were the effects of this co-construction of hope on users' experiences of hoping?

## Theory

There is a long tradition in the social sciences of considering how emotions are not merely feelings that happen to or within us but also feelings that we work upon. Sartre (1962, p. 12) suggested a person's agency over emotions as well as how they are “organised” by wider social forces. In related existentialist and phenomenological traditions, Kierkegaard (1957) and Schutz (1967) refer to ways in which our subjective gaze and attentiveness may be oriented towards, or away from, particular phenomena with important implications for our experiences of thinking and feeling.

Hope can be usefully understood within this tradition in that through a focus on a possible outcome or entity located in the future, we can bracket aside or “look past” (Brown and de Graaf, 2013, p. 554) difficulties, fears, and vulnerabilities in the present. In this sense, hope represents an important and potentially powerful mode of coping amidst the vulnerability of the present and uncertain future. Although these same tendencies also give hope a “dark side” (Brown, 2011), hopes can be manufactured – manipulatively and/or desperately – which would lead to some or many individuals pursuing or enduring conditions which are problematic and unjust (van Dantzig and de Swaan, 1978; Simpson, 2004).

However, judging when hopes are “appropriate” or “reasonable” and when they are inflated and misleading is not straightforward. As already noted, hopes inherently involve a tension between a desired possible and an expected probable (Simpson, 2004), but who gets to legislate what is “probable” remains unclear. Social science literature on risk (as a probabilistic tool for considering probable futures) notes that assessments of the probable futures tend to reflect the values and epistemic hierarchies around the powerful centre, at the expense of those on social peripheries (Douglas and Calvez, 1990). Nik Brown's (2015) work on hope and the science behind hope scales (measurement tools for hopes) makes a similar argument by which an orientation towards hope in cancer care is analysed as having its roots within social, psychoanalytic, and medical science developments in the inter-war and post-war periods of the twenteeth century in western Europe and north America. Within these contexts which had been characterised by (wartime) adversity and hardship, hope had come to be “expressed as an essential moral property of the person rooted firmly in the problem of wartime morale and civic determinedness” (Brown, 2015, p. 124), that is, as a context-specific norm for how one ought to feel, related to a particular moral framing.

We can consider the historical shifts in the framing of recovery in relation to mental health problems (briefly noted earlier) and the role and the orientation of hope within this framework, similarly as a representing a norm of how one should feel and act, rooted in a wider framing which develops at a particular moment in time (Ramon et al., 2007). Petersen (2015) points towards the earlier, more theologically oriented works on hope by Bloch (1986) and Fromm (1968), in denoting the activating role of hope, one which may be internalised as an “inner-readiness” (Petersen, 2015, p. 6), before going on to note the ideological and consumerist orientations of this combination of desire and expectation. Broader ideological tendencies may therefore be internalised through socio-political processes of hoping which, in turn, generate underlying dispositions towards acting in particular ways. In this sense, it may be useful to conceptualise hope as an emotion (Simpson, 2004), in terms of its emotional resonance (Simpson, 2004) but also in terms of Hochschild's (1979) work on framing and feelings rules. Although it is very difficult to delineate what is a reasonable or unreasonable hope in a more prescriptive sense, we can point towards evidence of “feeling rules” (Hochschild, 1979), within specific social settings, whereby norms exist regarding how we are expected to be hopeful in particular ways (Delvecchio Good, 2001; Brown, 2015); or indeed where hoping may be understood as, or commonly feel, inappropriate.

Although there is some ambiguity about how emotional norms or “feeling rules” relate to dominant ways of thinking (“framings”), Hochschild (1979) shows us how such norms for how we should feel can change across different times and spaces and, importantly, how these emotions should be understood in terms of interactions with others. As Black (2011), following Hochschild, has noted, vulnerable people may often work on “their own emotions in order to manage the emotions and responses of others, so that their own subsequent emotions could be further managed. In other words… managing emotion “by the self upon the self, by the self upon others, and by others upon oneself” (Hochschild, 1979, p. 562)” (Black, 2011, p. 188).

Such an attentiveness to dynamic emotional management, in dyadic interactions between patients and professionals, has been less thoroughly addressed in the medical sociological literature. Work has tended to focus more on the emotional labour of the professionals (e.g. Cottingham, 2017) or on the emotional features of illness management and narratives (see important work on hope and despair by Nowakowski, 2016), but less on how these interweave. Nevertheless, this literature on care and emotions gives us several important insights of relevance to our analysis of hope and its management, such as Cottingham's (2017, p. 272–273) call for attentiveness to “aspects of emotion that continue to appear natural and unintentional—operating in tandem with the conscious work of emotion management”.

This idea of more conscious and more taken-for-granted/non-deliberate approaches in care is of particular relevance in psychiatric care contexts where questions of risk, freedom, and capacity are often present. Driessen et al. (2017) study is one important example of where emotions, or in their case “wanting”, is analysed from an interactionist and socio-materialist perspective. These authors refer to the “will-work” carried out in dementia-oriented care homes whereby caregivers work to align carer and patient “desires” (p. 37). Driessen et al. (2017) stress in their framework that “wanting” is above all an “outcome of interaction” (p. 34), but various institutional and professional understandings of risk, freedom, and notions of what is “appropriate”, alongside the physical materiality and architectural layout of the care home, nevertheless underpin and limit what is negotiable (see Sellerberg, 1991). This recent work on caring in health and mental healthcare contexts illuminates the agency to negotiate emotions amidst specific contexts but that wider socio-structural framings are powerful and insidious.

Our findings on processes of hoping can be usefully explored and understood in these terms of feeling and framing rules and of managing emotions of the self and of others, as located within broader socio-political environments (Delvecchio Good, 2001; Petersen, 2015) and histories (Brown, 2015).

## Methods

### Design and context

Within one local mental healthcare organisation in southern England, three contrasting psychosis service settings were purposively selected as sub-cases, with each involving different configurations of vulnerability, uncertainty, and future possibilities: an early intervention service (working with young people aged 14–35 for up to 3 years), an assertive outreach team (this is an approach common in the UK whereby “assertive outreach” refers to seeking to maintain regular contact with individuals who are assessed to be especially vulnerable, who are deemed to pose a risk to themselves or others, and who are liable to avoid contact or rapidly disengage with services), and a more standard community mental health team. Psychosis services can be seen as constituting an extreme case (Miles and Huberman, 1994) for exploring processes, such as hope, by which service users and professionals may experience vulnerability and uncertainty in heightened form within these care contexts. This can render more explicit various taken-for-granted processes pertaining to how hope, or the hopelessness sometimes associated with mental distress, is shaped and managed. The research was conducted in 2009–2010.

### Sampling and participants

Inclusion criteria were all adult service users being treated by these psychosis services, and we worked closely with services to ensure that service users were contacted when they were not at a more vulnerable point in their illness trajectory. Initial plans for recruiting eight service users per service (*n* = 24) were unsuccessful. Despite eventually contacting 158 users through letters distributed through users' services, only eight service users were accessed overall (see Table 1 for participant characteristics). Although we recognise the limitations of the sample, which was less varied than we had hoped for (especially regarding race and ethnicity) and the tendency towards sample bias given the scale of non-response, this is a reasonable sample size for a segment of study within a phenomenological tradition (Smith and Osborn, 2003). Our prime focus was on the depth of interviews, which were successful in unearthing the important insights on hope reported below.

**Table 1:** Participant characteristics.

**Type of service**	**Service users**	**Professionals**	**Service managers**
Early intervention	2	4 [consultant, assistant psychologist, social worker, community psychiatric nurse (CPN)]	1
Assertive outreach	1	3 (consultant, social worker, CPN)	1
Standard community	5 (+1 carer)	3 (consultant, social worker, CPN)	1

The chaplain worked for overarching organisation and not with any one team.

Given the enforced distance between researchers and the non-respondents, it is difficult to account for such a low response rate although low levels of trust are one possible explanation and indeed our interviews with service users also reflected care contexts characterised by low levels of trust. Despite the small numbers, user participants represented diverse backgrounds and experiences (mean duration of contact with services = 15.9 years; SD = 12.4), including men and women (4 and 4), age (from 25 to 67), educational background (from leaving school at 16 to post-graduate study and increments in between), and economic activity (out of work; voluntary work; paid part-time work; retired). Small samples are less problematic within phenomenological studies where emphasis is placed on the depth of analysis of experiences and sense-making rather than broader patterns (Smith and Osborn, 2003).

Ten professionals were recruited via letters distributed through services, out of 12 contacted. We purposively selected a range of professional roles and levels of experience, including the clinical lead (consultant), one social worker, and one community psychiatric nurse within each team, as well as an assistant psychologist in one service. These participants had varying experiences in providing mental healthcare (mean duration working in mental health services = 16.1 years, SD = 10.6). The three service managers were also interviewed, as well as one carer and one chaplain.

We obtained written and oral consent, with participants assured of confidentiality and that participation was voluntary, meaning they could withdraw at any point. The project was carried out with local health service ethics and research governance approval.

### Qualitative interviews

In-depth, semi-structured interviews were carried out by one of two researchers, each experienced in interviewing professionals and vulnerable individuals. Interviews lasted between 45 min and 2 h for service users and carer, and 30 min to 1.5 h with professionals, managers, and chaplain. Service-user interviews began with a more narrative format, beginning with first contact with services, before asking participants to reflect on meaning and meaning-making amidst more positive and negative experiences via various themes (see Table 2). Due to time constraints and foci upon multiple relations and experiences, staff interviews were more thematically structured (see Table 3). Although hope was not specifically asked about, our interview approach was iterative and the underlying framework behind the research was sensitised by literature which notes a range of coping processes amidst vulnerability and uncertainty, including hope (e.g. Zinn, 2008). In later interviews, when participants did raise the theme of hope, we were prepared to probe the participants' experiences in relation to hope, to further clarify its meaning and social functioning.

**Table 2:** Topic list for in-depth semi-structured interviews with service users.

*Introduction* • Clarification of research and interview purpose • Reminder regarding voluntary participation, anonymity, and confidentiality • Demographic questions *Condition and experiences* • First contact with services, experiences then and since• Daily impact, experiences of uncertainty, coping, sources of support and treatment, helpfulness of different sources *Help, treatment and contact with services*• Approachability of services – obstacles, uncertainty, vulnerability • Views of services, sources of information on services • Interactions with different professionals and services – relative helpfulness, differences in experiences • Communication and openness with different professionals • Experiences of different treatments and medication • Understanding of medication and quality of information from professionals • Different levels of trust in different professionals *Trust* • Experiences where trust easier or more difficult – reasons for this • Impact of trust on relations with professionals • Impact of trust on contact with services, disclosure, and medication use General views of the healthcare system and mental health services *Closing of interview*• Reflection upon interview • Opportunity to ask questions and to add further ideas or thoughts not covered

**Table 3:** Topic list for in-depth semi-structured interviews with professionals.

*Introduction* • Clarification of research and interview purpose • Reminder regarding voluntary participation, anonymity, and confidentiality • Demographic questions *Working with service users – uncertainty* • Challenges of working in psychosis services • Necessary skills, attributes, and requirements • Dealing with different uncertainties *Relating to the service user and trust* • Nature and quality of relations with service users • Presence and role of trust • Trust building and changes over time *Working with risk and vulnerability* • Risk assessment within professional work • Changes to working with risk • Vulnerabilities in assessing risk *Teamwork* • Extent of teamwork within professional duties • Nature of more effective working relations • Information exchange and communication *Working with managers* • Impact of and interactions with managers • Relations with and views of managers • Trust between professionals and managers *Closing of interview* • Opportunity to ask questions and to add further ideas or thoughts not covered

### Analysis

Interviews and analysis were informed by interpretative phenomenological approaches. Detailed exploration of ongoing, interactive processes of sense-making and expectation construction amidst uncertainty – as these were shaped by social biographies and accumulated “taken-for-granted” assumptions – was central (Schutz, 1967; Smith and Osborn, 2003). Data coding therefore combined more open approaches, where data were first broken down and related fragments were continually compared with one another, with the latter more selective phases.

Hope emerged as an important theme within open coding. The concept was then returned to via a secondary analysis where we aimed to understand specific references to hope phenomenologically, in relation to the intersubjectivity, bracketing-off, sense-making processes, and lifeworlds of the participants (Schutz, 1967; Smith and Osborn, 2003). Different layers of theme-generating work thus applied phenomenological considerations (Schutz, 1967) as a “sensitising” approach (Blumer, 1954).

Initial analyses were further refined through ongoing comparison and discussion amongst two different coders, paying special attention to common interpretations by the participants as well as to rich cases which differ from broader theoretical patterns (Smith and Osborn, 2003; Lindseth and Norberg, 2021). We critically discussed emerging themes from this coding process with social science research colleagues and experienced clinician-researchers, in seeking to further enhance the reflexivity and internal validity of the analysis.

## Findings

Hope was a common and often important theme within service-user and professional participants' accounts. Our analysis is presented under headings pertaining to overarching subthemes developed during the analysis.

### Professionals' emphasis on the importance of managing hopes

Notions of hope were common within and across the accounts of service users (*n* = 7 of 8) as well as those of professionals (*n* = 6 of 10). Some professionals did not mention hope at all when describing their work with service users amidst vulnerability and uncertainty, whereas others referred to managing hope as a key concern in their work:

Psychologist: *For me I think that's almost part of the ethos of the service really, is to have that…maintaining some hope. Because sometimes people do get better and I think that message… I think in those early days, perhaps it would be helpful [for service-users] to hear that people* do *get better from this*.

For this psychologist, hope was not only something relevant to their work but a rather central consideration to the service. Imagined futures are the foundation of hope – in contrast to trust which is grounded in interpersonal interactions of the present and past (Möllering, 2001; Brown and de Graaf, 2013) – hence the ongoing envisaging of positive possible outcomes was vital to the interwoven processes of desire and imagination (Simpson, 2004) by which hopes were fostered. Encouraging a consideration of possible positive futures was seen, above, as important but professional participants also tended to stress a need to “balance” this with “being realistic”, as the social worker below suggested:

Social worker 3:*…and I suppose that's where people skills come in, it's keeping that hope alive but also being realistic at the same time – and I think that's the biggy* [the big challenge] –* it's the hardest balance. It's kind of allowing people to know what could happen, and the avenues [of] whether the illness will continue; whether it will just be a one off episode and what the treatment options are; trying to give a kind of positive message and not be too kind of negative*.

As we see above, alongside maintaining hopes, this social worker spoke of their role in helping service users to frame their futures “realistically” (Wiles et al., 2008). Implicit here were ontological assumptions pertaining to a “reality” and epistemic assumptions regarding the capacity of mental health professionals to evaluate an individual's likely future in light of probabilistic knowledge of outcomes across populations. Despite the challenges in applying probabilistic knowledge to individual cases, the population-level knowledge nevertheless serves as a strong basis for professionals' imagining, and in some cases imposing, of patients' futures.

Several professionals described techniques of managing expectations from the outset and limiting service users' envisaged possibilities. This partly reflected what was deemed (im)possible or likely, but also implied negative past encounters that they were trying not to repeat:

Chaplain: *Sometimes you have to get in there within the first 5 minutes and say, “before we go any further, please, you ought to know that I can't just zap your… you know”. “If you're saying “I've got a demon, get rid of my demon”, I can't just do that”. And so you have to set out the boundaries very quickly, which sometimes upset them, but it's a matter of holding on to them so that you're able then to work with them over a number of occasions*.

As noted here, imperatives of managing expectations downwards – to purposively and explicitly frame or “bracket” (Brown, 2009) the user's possible futures within certain limits – were commonly referred to. In the case above, blind trust, or faith, in the capabilities of a person, the chaplain, was challenged as a means of limiting hopes in a specific outcome. Achieving this “balanced” hope was considered vital to avoid, on the one hand, a loss of hope and corresponding coping and motivation and, on the other, a slippage from hope towards a position where uncertainty and negative eventualities were ignored and expectations became “too high”.

Some professionals (*n* = 5) thus described actively managing the hopes of service users, drawing upon their communication skills and experience to do so, while a few professionals also described managing their own hopes for service users. Given the vulnerability and uncertainty they faced, motivation and coping through hope was important. This was especially mentioned by professionals (*n* = 3) from one particular service where hope was seemingly discussed and made explicit within the team. As with service users, too much hope could potentially lead to disappointment and frustration for professionals:

Consultant psychologist 1: *I think we're in a kind of later phase of development now but initially…and that initial phase lasted quite a while…initially I think we idealised the service-user group so that we took hope to a ridiculous extent, almost like a belief that everybody could be made better. It's an unrealistic expectation*.

This same senior clinician went on to describe how the team were adjusting, or managing, its hopes towards building a more “realistic” outlook which would, in turn, reduce disappointment while maintaining the drive of the team. These considerations were also described as relevant to the recruitment of new professionals.

*So more recently I think we're responding to that, the strain of working with this service user group, by just rowing back a bit and trying to find that balance between having realistic expectations and still… being motivated to do the best for the service user group… [When recruiting], we're quite keen on people who have already done quite a bit of work with people with psychosis; and I think that's probably to weed out some of that idealism really*.

Some professionals could therefore be understood as managing hopes in a collective sense for the sake of colleagues, as well as for service users and indeed for themselves (Hochschild, 1979). This management of hopes for others was chiefly conducted by challenging the framing of others' thinking (Black, 2011). Recovery, as a concept in mental health care, has encouraged a hopeful approach to supporting and *coping* amid chronic health problems. Apparent in the excerpt above was a reframing of hope towards this recovery approach, away from notions of a complete absence of psychosis symptoms.

### Service-users' balancing of hopes and tensions therein

Whereas hope was absent from some professionals' narratives, only one service user did not refer to hope as significant within ongoing experiences and coping. This service user was by far the most stable and “recovered” of the eight participants in the study. Within narratives often characterised by heightened levels of uncertainty and vulnerability, the other seven participants referred to hope as very important in various senses, especially for identity, motivation, and coping:

Service-user (SU) 1 – schizophrenia diagnosis – man in his 30s: *Yeah…You know, that is the light at the end of the tunnel…Because you…you can only imagine…you know…how could you have a girlfriend or…no-one would understand why these Assertive Outreach Team were coming around and no-one would find it acceptable…*

For this service user, the “light at the end of the tunnel” was leaving the care of the assertive outreach service for a standard community team (or even a General Practitioner as a longer-term hope), within which a more “normal” life and social relations could develop. Hopes for improved coping and independence were recurring features of living with severe mental health problems, reflecting the concept of recovery, though descriptions of hopes varied between more modest and higher hopes:

SU5 – schizophrenia diagnosis – woman, age 60s: *And the thing is, [what] I find in these later years, is negative thinking is no good. You must think positive. If I think positive then I'm on the way, well on the way to grabbing that goal that I want so badly. So I'm all [set] to go and get that goal…and I hope one day to be able to say that I can go out on my own, like I'm learning to now, slowly…I don't dash at it at once…because I know that I just take it easy and feel my way*.

Service users' accounts often involved an interweaving of envisaging and desire, which has been described as a characteristic of hoping (Simpson, 2004). As with a number of accounts, the service user above referred to managing her hopes upwards – towards thinking positively. Yet this managing of hopes had seemingly also been shaped by three decades of experiences with services and several hospitalisations during that time – thus at the end of this quotation, she referred to not expecting too much too soon.

Acute experiences involving hospitalisation were, for various participants, narrated in terms of hopes and expectations which had been too high, with these resulting in negative outcomes:

SU8 – bi-polar diagnosis – woman, age 30s: *I think the key was I couldn't accept that this was a condition that was longer term…I mean it's partly to do with the kind of weakness concept but I wanted to think that I'd beaten it, full stop, and there used to be quite long periods between relapses so I'd think that I'd made it out to the other side*.

Apparent in this account were complex interactions between denial, stigma, insight, hope, and coping with illness (Lysaker et al., 2007). These tensions were important to the managing of expectations and relating these to different desires was central to what it meant to manage one's hopes. As this same service user continued:

SU8:*…there are times when I cut down my medication a little bit but I don't stop it…I just feel there's so many positive things in my life now that I've struggled so much with, especially my job, that I'd be so foolish to throw them away…*

While describing the quality of support she had received, this user emphasised that the restructuring of illness conceptions and related aspirations were not a direct result of interactions with professionals, but where she had learned herself to not be too hopeful, or rather to hope for coping rather than life beyond medicines and mental health services:

SU8: *A lot of the – I won't say – “resignation”, has come from me really*.

In contrast, some other service users' narratives pointed to the great influence of professionals in shaping their hopes – sometimes purposefully (as noted in the preceding section) and sometimes less wittingly, as apparent here:

SU7 – schizophrenia diagnosis – man, age 33: *I'm hoping to get over it [schizophrenia] at some point and be able to have a normal life…It's worrying sometimes…My consultant, that I was under at the hospital I was recently in, told me that I'd probably have to be on medication the rest of my life and I would never recover fully, that I would probably be ill forever, which was a bit like, you know: “I don't want to be told that!” But [earlier] the other doctor there told me that I'd have to be on the medication for a year at least and that… gave me a much more positive outlook*.

While both clinicians referred to here were seemingly communicating their professional opinion, interpretations made from these contrasting two prognoses – offered by two psychiatrists within the same in-patient service, within a short period of time – had quite contrasting impacts on this service user's emotional orientation towards the future and, consequently, to experiences in the present. Although not actually conflicting, the approach which avoided negative longer-term prognoses and focused on the shorter-term prognoses was seemingly more effective in equipping the user with motivation. This latter communicative approach would also seem to acknowledge the uncertainty around longer-term psychiatric prognoses.

### The co-construction of hope and its consequences

As was apparent in the latter quote in the preceding section, the more or less witting management of service users' expectations by professionals could place significant strain upon hopes, leading in some instances to hopes being significantly undermined, despite what was desirable or imaginable. The same service user went on to describe the negative effects of this loss of hope:

SU 7:*…that [being told I would never get better] haunts me now. So, you know, it would be nice just to be told that there's chances of things happening [for the better]*.

Here this same service user described a vulnerability following the undermining of his hope. Although this participant did not attempt to give a specific, word-by-word, account of what was said to him amid the more negative prognosis, his recollected interpretation seemed to have led to a loss of hope in improved and more effective coping, as well as a loss of hope of a symptom-free life.

In this excerpt, we see evidence of different approaches by professionals as well as the agency of the service user in interpreting and framing communications in particular ways. Although the quote above shows the negative impact of the communication in “haunting” this service user, he also went on to reflect upon how a relative lack of trust in this particular psychiatrist enabled the (partial) insulation of his understanding of himself from the negative prognosis:

SU7: *Yeah. I had a doctor 10 years ago and I think he spent a lot of time to get to know me and he diagnosed me as having something else and I…I still think that he* [that earlier doctor] *was right and I'm not so sure about this one*.

Varying levels of (dis)trust in the professional could therefore lead service users to accept or reject professionals' opinions regarding diagnoses and, in turn, to focus upon or “bracket off” the forecasted futures connected to these (Schutz, 1967; Möllering, 2001). Social processes around hope in outcomes and trust in professionals could therefore be seen as complexly interwoven (Brown et al., 2014) and involving multiple layers of agency (Brown, 2009).

Professional views regarding “realistic” expectations, alongside the service user's own experiences, could in some senses limit hopes-as-expectations (in the probable) and yet hopes-as-desires (in the possible) were nevertheless considered and emphasised within users' outlooks:

SU5*:She [my psychiatrist] will give me the all clear one day when I'm fit enough to be left out on [an] even keel with no problems of mental health again. So I'm hoping that by helping myself, and…[through] further involvement with the mental health people…*

This more hopeful perspective of complete recovery contrasted markedly with the modest hopes expressed by the same service user, as quoted at the start of the preceding section. Tensions between these two segments of the same interview narrative, and between these latter hopes and the service-users' chronic struggles with severe mental health problems for more than three decades, indicated some important features concerning the nature of hoping, as were common across many of the narratives: (a) hope involved inherent tensions between desires and expectations, and (b) hoping was built upon imagined futures and yet partially constrained by lived pasts (Wiles et al., 2008).

These tensions and ambivalences were common, especially within accounts of service users. It was service users' involvement with services which seemingly shaped such ambivalences towards the future, whereby services represented a source or focus of hope, but also an authority which often emphasised the chronicity of their condition:

SU2 – awaiting-diagnosis – woman, age 26: *I don't want to, you know, count my chickens before they hatch as such…I need a diagnosis and for myself really, because then I can put my finger on it and go “well that's what it is” and… and we can all do what we can to get me better*.

This service user had had a number of very difficult experiences and relations involving mental health services in the past. Nevertheless, the outcome of finally receiving a bi-polar diagnosis became the focus of her hopes, which in turn acted as the basis for further hopes in her condition improving, accordingly motivating her in the present. Importantly though, she went on to qualify these hopes, as she had been encouraged to do by her main support worker in a framing consonant with a recovery model approach:

*SU2:… But obviously if it is bi-polar there's no cure for bi-polar, it's just how I'm gonna deal with it from day to day*.

This ambivalence in hoping here, as apparent in a desire for a diagnosis alongside a recognition of the long-term challenges connected to this diagnosis, was similarly apparent in the account of another service user who described past experiences with a particular mental health service as offering him little help or hope. Yet, this same service also represented for him the only imaginable possibility of dealing with the difficulties he was facing:

SU6 – no diagnosis – man, age 25: *And the [service] discharged me…around September [year] and said, “if there's ever a problem again come back to us”. I came back to them, I don't know why, all I know is if there's something that can be done, it has to be done, so then I'm back with [the service]. I've an initial appointment [with a psychologist]…on Monday morning. It's a new avenue, I hate to say “a stab in the dark” but it's…it's…I hope it's gonna be the right thing*.

By giving this man the possibility to return to the service, a hope-as-desire in a solution was kept alive, even though services had done little to generate positive expectations of effective treatment in this user's past experiences. As with SU2 (directly preceding), this service user's hopes-as-desires and hopes-as-expectations were both shaped by his interactions with a service. The tension between these two future orientations can therefore also be understood as a product of co-construction processes between users and services. Such tensions were very much a feature of service-users' hoping narratives, as strongly apparent within six of the eight users' narratives discussed above.

## Discussion

Such awkward tensions (as noted directly above), alongside the way interactions shape these various features of hoping, are central to grasping experiences of hoping yet seldom considered in the literature about hoping amid mental health problems. Literature reviews, moreover, note a lack of clear consideration as to what hope-oriented mental health and social care might look like in practice, alongside a continuing ambiguity around the concept of hope amid mental health and illness (Cutcliffe and Koehn, 2007; Schrank et al., 2008; Heller, 2014). The central aim of our analysis above has been to explore the co-construction of hope amidst interactions between service users and professionals, as well as to consider the influence of these co-constructions on users' experiences of hoping.

That service users commonly and spontaneously emphasised hope – as enabling coping alongside a more positive and motivated sense-of-self in the present (Repper and Perkins, 2003) – indicated its salience. The size and nature of our sample render the transferability of our findings to other contexts highly tentative, yet that professionals referred less commonly to hope than service users may tell us as much about contrasting dominant “vocabularies” (Mills, 1940), alternative means for pursuing coping and control amidst uncertainty, and the nature of late-modern mental healthcare. The emergence of hope as one such vocabulary amidst mental health services where “recovery” has become an important “framing rule” (Hochschild, 1979), as well as the way this hope was then often managed in relation to probabilistic and risk-dominated framings of mental health problems, tells us much about the ideological features which Hochschild (1979, p. 557) sees as the other side of the “feeling rules” coin.

Indeed whereas Hochschild has been criticised for a lack of specificity when it comes to what ideology means as a basis for framing rules, in our study we saw how diagnoses, prognoses, past direct experiences of service users, risk-related policy, and notions of recovery could all be considered as important sources of framing from which certain norms of reasonable hope were derived. These interactive processes can, in turn, be located within professionals' understandings and framings shaped by current scientific evidence, their own past individual experiences, and broader policy narratives such as those emphasising specific meanings of recovery and hope's role within this. Such a discursive regime of hoping can, in turn, be located historically within particular socio-political, scientific, and health system regimes (see our discussion in the introduction following Ramon et al., 2007; Brown, 2015). In this sense, hope-related imaginaries involve multiple layers of lifeworlds in which interactions and identities are embedded (Schutz, 1967; Habermas, 1987; Delvecchio Good, 2001).

Diagnosis and prognosis in psychiatry are notoriously complex and uncertain. This uncertainty opens up affective aspirational spaces for hoping yet these possibilities for imagining otherwise could sit in awkward tension with common assumptions regarding the chronic nature of many mental health conditions (as Service User 2 reflected in the final data section). The relative power of mental health professionals, in contrast to vulnerable and stigmatised service users, also helps us understand the dynamics in which views of appropriate framings and feelings about the future could be imposed (Delvecchio Good, 2001).

A particular attentiveness towards hope within one local team of professionals suggested to us that specific senior professionals may cultivate a hope awareness within a professional team, leading to a more conscious management of framing and feeling towards the future “by the self upon the self, by the self upon others, and by others upon oneself” (Hochschild, 1979, p. 562). However, there was little evidence that this deliberative “working” on hope was common. Professionals could also, unwittingly, inflate their own hopes and those of their colleagues in ways which were described as problematic for longer-term motivation and coping within service teams.

A similar picture emerged within service-user accounts of how their hopes were more or less inadvertently shaped during interactions and experiences with services. A few professionals described, and were interpreted by users as, managing expectations. However, professionals also raised or undermined expectations and desires unintentionally. Indeed, from a phenomenological perspective, this is inevitable given the active role of the interpreter in giving meaning to the utterances of others (Brown, 2009). Service users referred to managing their own hopes as well as the impact of interactions with professionals on their hopes. Hopes-as-desires and hopes-as-expectations were both discernible within accounts, with experiences of *hoping* best conceptualised as living amidst a tension between these two dimensions of hope (Wiles et al., 2008; Brown and de Graaf, 2013).

This conceptualisation of hope and its co-construction contrasts markedly with certain studies depicting hope simply as an enduring and underlying trait (Wiles et al., 2008). Users were still able to exercise agency over their hopes amidst these co-constructions, for example, by disregarding (or bracketing) the views of a professional who was not well trusted or by continuing to focus on desires regardless of a reshaping of their “expectations”. Nevertheless, a few service users referred to unrealistic or undermined hopes and how this had left them exceedingly vulnerable (Chadwick, 1997).

Present within the literature are understandings of specific interventions and their impact on hope, often in relation to recovery (Schrank et al., 2012). However, with these studies typically focusing on hope as a purely positive phenomenon, little detailed analysis exists around the successful management of *balanced* hopes. Professionals referred to positively managing expectations downwards in some cases or maintaining hopes within certain parameters. This deliberate intervention by professionals suggests a paternalism that may be at odds with the understandings of the patient empowerment basis of hope in recovery models. Communicating more explicitly about differences in expectations and desires may help overcome some of these difficulties (Wiles et al., 2008), but tensions between desires and expectations would seem inherent to the nature of hoping (Simpson, 2004), especially within chronic and debilitating conditions involving psychosis.

### Methodological issues

Although our selections of services and staff were purposive, the participation of service users was especially low. However, our phenomenological approach was more concerned with the in-depth exploration of the meaning and experience of hope and its co-construction, rather than seeking to generalise across users and services (Smith and Osborn, 2003). Hope was not enquired about specifically and, despite probing when raised, there was a lack of consistent questioning around hope. This approach may have facilitated certain findings which contrast with existing studies of hope in mental healthcare settings, for example, in our illuminating of the ambiguity and multiplicity of hopes and the tensions existing between more modest and elevated hopes.

### Conclusion and implications

The role of hope is increasingly acknowledged through the prominence of “recovery” within mental healthcare services, yet the task of managing hopes is far from straightforward (Heller, 2014) – involving a highly sensitive balance or tension between optimism and realism, desires, and expectations. Enduring uncertainty around longer-term outcomes, alongside poverty and violence experienced by some users in everyday life, renders possibilities for “achieving ordinary lives” (Bertolote and McGorry, 2005) precarious. A dark side accordingly lurks where motivation and coping are enacted via inflated hopes. Professionals accordingly need to become more alert to the way they knowingly or unwittingly manage the hopes of service users in their care. Professionals have limited control over how the meaning of their words and other forms of communication are interpreted (Schutz, 1967), posing a challenge to managing hope. However, this challenge may be partially addressed through improved communication, attentive listening, and open discussions with service users with the aim of managing inherent tensions between hope-as-expectations and hope-as-desires, to achieve a “balanced” hope, responsive to changes in the service user's relationship to their condition.

Professionals and managers also need to be sensitive to the hopes and expectations of colleagues – similarly managing these and avoiding inflated hopes, which may lead to burnout, while maintaining an atmosphere of “realistic” positivity and hopefulness as a basis of a dynamic, cohesive, and motivated professional teams. Open and candid discussion may, again, be vital to such management of hopes. Our findings suggest that training and recruitment can also be relevant. Above all, it would seem that every professional plays an important role, consciously or not, in managing their own hopes as well as those of others.

## Data availability statement

The datasets presented in this article are not readily available because we quote from our in-depth qualitative interview data in ways which minimise identifiability. The ethics clearance and participant consent was given on the basis that we would not share full interviews with other parties. Sharing or making the full data set available would breach the trust and ethical understandings on which our research was based. Requests for information about the data sets should be directed to p.r.brown@uva.nl.

## Ethics statement

The studies involving humans were approved by the Kent NHS Research Ethics Committee. The studies were conducted in accordance with the local legislation and institutional requirements. The participants provided their written informed consent to participate in this study.

## Author contributions

PB: Conceptualization, Data curation, Formal analysis, Funding acquisition, Investigation, Methodology, Validation, Writing – original draft, Writing – review & editing. AS: Conceptualization, Supervision, Writing – review & editing. MC: Conceptualization, Funding acquisition, Methodology, Writing – review & editing, Validation.
